# Molecular Screening and Characterization of Canine Coronavirus Types I and II Strains from Domestic Dogs in Southern Italy, 2019–2021

**DOI:** 10.1155/2024/7272785

**Published:** 2024-04-18

**Authors:** Francesco Mira, Giorgia Schirò, Gianvito Lanave, Gabriele Chiaramonte, Marta Canuti, Elisabetta Giudice, Paolo Capozza, Vincenzo Randazzo, Francesco Antoci, Donato Antonio Raele, Domenico Vicari, Annalisa Guercio, Nicola Decaro, Giuseppa Purpari

**Affiliations:** ^1^Istituto Zooprofilattico Sperimentale della Sicilia “A. Mirri”, Via Gino Marinuzzi 3, Palermo 90129, Italy; ^2^Department of Veterinary Sciences, University of Messina, Viale Palatucci, Messina 98168, Italy; ^3^Department of Veterinary Medicine, University of Bari, Strada Provinciale per Casamassima Km 3, Valenzano, Bari 70010, Italy; ^4^Department of Pathophysiology and Transplantation, Università degli Studi di Milano, Via Francesco Sforza 35, Milano 20122, Italy; ^5^Centre for Multidisciplinary Research in Health Science (MACH), Coordinate Research Centre EpiSoMI (Epidemiology and Molecular Surveillance of Infections), Università degli Studi di Milano, Milan, Italy; ^6^Istituto Zooprofilattico Sperimentale della Puglia e della Basilicata, Via Manfredonia 20, Foggia 71121, Italy

## Abstract

Canine coronavirus (CCoV) is a common agent of gastroenteritis in dogs, although some variants have been found associated with systemic and often fatal diseases. Distinct genotypes (CCoV-I and CCoV-II) and subgenotypes (CCoV-IIa and CCoV-IIb) are worldwide distributed. In Italy, CCoV infections have been occasionally evaluated, but information about the molecular epidemiology and the genomic features of currently circulating strains is limited. This study reports the detection and molecular characterization of CCoV strains from samples collected from 284 dogs in Italy between 2019 and 2021. CCoV RNA was detected in 39 (13.7%) dogs, as a single viral agent (5 animals, 12.8%) or with other viral pathogens (canine parvovirus types 2a/2b/2c; canine adenovirus type 1; norovirus GIV.2) (34 animals, 87.2%). A total of 48 CCoV strains were detected either alone (CCoV-I: 51.3%, CCoV-IIa: 20.5%) or in copresence (CCoV-I and CCoV-IIa, 23.1%); surprisingly, CCoV-IIb was not identified in this study. Five clusters of CCoV-I were detected, and their spike gene sequences showed the highest nucleotide identities with CCoV-I strains collected from Greece in 2008/2009 and from China in 2021. CCoV-IIa spike gene sequences (three variants) had the highest nucleotide identities with CCoV-IIa strains collected in Greece in 2008/2009 and in Italy in 2009/2011. Given the high CCoV diversity and the variable pathogenicity potential, we underline the need of further surveillance studies to increase our understanding of the epidemiology and evolution of these viruses.

## 1. Introduction

Canine coronavirus (CCoV) is taxonomically included in the species *Alphacoronavirus 1* (family *Coronaviridae*, genus *Alphacoronavirus*, subgenus *Tegacovirus*) along with other coronaviruses of domestic mammals, such as feline coronavirus types I and II (FCoV-I and FCoV-II), transmissible gastroenteritis virus (TGEV), and porcine respiratory coronavirus (PRCoV) [1]. Coronaviruses include large, enveloped, single-stranded, positive-sense RNA viruses. The CCoV genome consists of an approximately 30 kb-nucleotide (nt) long RNA molecule containing different open reading frames (ORFs). The 5′ two-thirds of the genome consist of two partially overlapping ORFs (ORF1a and ORF1b) containing the genetic information for nonstructural proteins, while the 3′ side contains ORFs encoding for the major structural proteins, including the spike (S), envelope (E), membrane (M), and nucleocapsid (N) proteins [2]. Among the structural proteins, the S glycoprotein is the most variable one, represents the major inducer of virus-neutralizing antibodies, and plays a role in the biology of CCoV as it induces both viral envelope-host cell membrane and cell-to-cell fusion [2]. The S protein contains a large ectodomain consisting of two subunits: the receptor-binding subunit S1 and the membrane-fusion subunit S2 [3]. Within the S1 subunit, a major domain known as the N-terminal domain (NTD), located in the first 300 amino acids (aa) of the subunit, is an important determinant of intestinal tropism in the closely related TGEV [4].

Based on the divergence in the S protein sequences, two distinct genotypes, named canine coronavirus type I (CCoV-I) and canine coronavirus type II (CCoV-II), have been reported worldwide [1]. During the course of CCoV evolution, new genetically divergent strains, some characterized by more pronounced pathogenic potential, have emerged through genetic drift or recombination [5]. Accordingly, two subgenotypes (CCoV-IIa and CCoV-IIb) have been reported [6].

CCoV was first described in 1971 in Germany [7], and to date, it appears to be enzootic worldwide causing, as a single viral agent or in coinfection with other canine viral enteropathogens, mild to moderate gastroenteritis in domestic dogs [8, 9]. Some variants are associated with systemic and often fatal disease [5]. Moreover, the detected sporadic evidence of CCoV in wild animals [10–13] posed questions on the risk for cross-species transmission.

Since the late 1990s, an increasing number of studies conducted in Italy focused their attention on the analysis of CCoV genomic variability and evolution, developing new diagnostic and genotyping tools that allowed to acquire knowledge on currently circulating strains and to evaluate their pathogenic potential [5, 14]. CCoV infections have been reported in Italy in both domestic dogs and wild carnivorans [15–19], but information on the molecular epidemiology and the genomic features of strains circulating in recent years is limited. This study reports the detection and molecular characterization of CCoV strains from samples recently collected from dogs in Italy. The aim of this study was to provide a more in-depth update on the epidemiological and molecular features of CCoVs identified in domestic dogs in Italy and to study them in the current epidemiological scenario by comparing them to strains identified in other countries.

## 2. Materials and Methods

### 2.1. Clinical Samples

This study utilized samples submitted to the Istituto Zooprofilattico Sperimentale della Sicilia “A. Mirri” (Italy) between January 2019 and December 2021 for diagnostic purposes. Samples from 284 owned (*n* = 125), shelter (*n* = 75), kennel (*n* = 10), imported (*n* = 2), or stray (*n* = 72) dogs with suspected viral enteritis were analysed. Samples from live dogs in shelters were collected at the time of admission (either for first aid cares or rehoming purposes) when gastroenteric clinical signs were observed. Samples included faeces or rectal swabs from 117 living animals and tissue samples (lungs, spleen, liver, intestine, mesenteric lymph nodes, and kidneys) from 167 dead dogs that were subjected to necropsy. Sample details are summarized in *Supplementary 1*.

### 2.2. Virus Screening

Rectal swab and organ homogenate solutions were obtained: briefly, samples were homogenized in a 10% w/v culture medium (Eagle's Minimum Essential Medium; Sigma–Aldrich®, Milan, Italy) containing 2% fetal bovine serum (EuroClone®, Milan, Italy) and an antibiotic and antimycotic solution and then centrifuged at 1,500x *g* for 10 min at 4°C. Total RNA and DNA were extracted from homogenates using the QIAamp Viral RNA Mini kit and the DNeasy Blood & Tissue kit (Qiagen S.p.A., Hilden, Germany), respectively, according to the manufacturer's instructions.

The presence of CCoV RNA was confirmed using the CCV1-CCV2 primer pair [14] targeting a fragment of the matrix (M) gene by reverse transcription (RT)-PCR. Reactions were carried out using the QIAGEN® OneStep RT-PCR kit (Qiagen S.p.A.) in a 50-*μ*l mix containing 1 *µ*l of each primer (20 *μ*M) (*Supplementary 2*) and 0.25 *μ*l Rnase inhibitor (40 U/*μ*l, Euroclone S.p.A., Pero, Italy) and using 2.5 *μ*l of RNA extract as input. The reaction was conducted under the following thermal conditions: 50°C for 30 min, 95°C for 15 min, followed by 40 cycles of 94°C for 60 s, 55°C for 60 s, 72°C for 60 s, and a final extension of 72°C for 10 min. Detection of CCoV RNA was confirmed by the Infectious Diseases Unit of the Department of Veterinary Medicine of Bari (Italy) by using a previously described quantitative real-time RT-PCR (RT-qPCR) assay [20].

Extracted DNA/RNA from all samples was also screened with a set of traditional or real-time (RT-)PCR assays (qPCR) for the detection of canine parvovirus type 2 (CPV-2), canine adenovirus types 1 and 2 (CAdV-1 and CAdV-2), canine distemper virus (CDV), norovirus (NoV), and rotavirus (RoV), with primer pairs and probes previously described [21–25].

### 2.3. CCoV Genotyping and Subgenotyping

For CCoV genotyping, a set of five primer pairs [26, 27] targeting fragments of the M and S genes were used in separate RT-PCR assays. Reactions were carried out using the QIAGEN® OneStep RT-PCR kit in a 25-*μ*l mix containing 1 *µ*l of each primer (20 *μ*M (*Supplementary 2*) and 0.25 *μ*l Rnase inhibitor (40 U/*μ*l) and using 2.5 *μ*l of RNA as input. Reactions were conducted under the same thermal conditions described above, except for the use of an annealing temperature of 50°C for the primer pair CEPol-1/TGSP-2. Two further qPCRs amplifying a fragment of the M gene, as described in Decaro et al. [28], were performed at the Department of Veterinary Medicine of Bari for the confirmation of CCoV-I and CCoV-II typing.

Samples that tested positive for CCoV-II were further subgenotyped in CCoV-IIa or CCoV-IIb through a traditional RT-PCR assay, using primer pairs 20179/INS-R-dg or 20179/174-268 targeting the S gene (*Supplementary 2*) as described by Decaro et al. [15]. Reactions were carried out with the SuperScript® IV One-Step RT-PCR system (Invitrogen srl, Waltham, USA) in a 25-*μ*l mix containing 0.5 *µ*l of each primer (50 *μ*M) 20179 and INS-R-dg or 174–268 (*Supplementary 2*) and using 2 *μ*l of RNA extract as input. The following thermal protocol was used: 50°C for 10 min, 98°C for 2 min, and 40 cycles of 98°C for 10 s, 55°C for 10 s, and 72°C for 30 s, with a final extension at 72°C for 5 min.

### 2.4. CCoV Sequence and Phylogenetic Analyses

All CCoV positive samples were subjected to two separate RT-PCRs targeting the 5′-end of the S gene with primer pairs published by Ntafis et al. [29], Decaro et al. [30], and Ma et al. [31] (*Supplementary 2*), using the SuperScript® IV One-Step RT-PCR system as described above.

Amplicons obtained from the assays described above (M and S genes) were purified with Illustra™ GFX™ PCR DNA and Gel Band Purification Kit (GE Healthcare Life Sciences) and submitted for direct Sanger sequencing to BMR Genomics srl (Padova, Italy). Sequences were assembled and analysed using Geneious Prime 2022.0.2 (Biomatters, San Diego, CA, USA) software. A sequence identity matrix was generated from each alignment (CCoV-I and CCoV-II) with BioEdit 7.2.5 [32]. The prediction of potential N-linked glycosylation sites was carried out using NetNGlyc 1.0 [33].

To evaluate the phylogenetic relationships between the analysed CCoVs and other strains retrieved from the GenBank database (accessed on January 24, 2023), phylogenetic trees based on partial M and S gene sequences were built using the best-fit model of nucleotide substitution (Tamura 3-parameter and general time reversible with gamma distribution) with MEGA X software [34] using the maximum-likelihood method with 1,000 bootstrap replicates.

Obtained sequences have been submitted to the DDBJ/EMBL/GenBank databases under accession numbers OQ565677–OQ565713 (gene M) and OQ565714–OQ565743 (gene S).

## 3. Results

### 3.1. CCoV Detection and Typing

CCoV RNA was detected in 39 (13.7%) out of 284 sampled dogs: CCoV was identified as a single viral agent in five dogs (12.8%) or with other viral pathogens in the other 34 dogs (87.2%). There was full concordance between RT-PCR and RT-qPCR results. Among other viruses, CPV-2 (all three CPV-2 variants, *Supplementary 1*) was the most frequently identified agent (33 dogs, 84.6%), while CAdV-1 (2 dogs, 5.1%) and NoV GIV.2 (three dogs, 7.6%) were less frequently observed (*Supplementary 1*). Almost all dogs that tested positive for CCoVs were puppies (84.6%, mostly approximately 2 months old), with the exception of one CCoV-I-positive dog (id. IZSSI_2019PA26638, 9 months old), two CCoV-IIa-positive dogs (IZSSI_2019PA27044, 5 months; IZSSI_2019PA34446, 11 months), and three CCoV-I/IIa-positive dogs (IZSSI_2019PA11822, 2 years old; IZSSI_2020PA45728, 5 years old; IZSSI_2021PA65740, 11 years old). Among living animals, mostly positive to at least two viruses, the proportion of dogs with a positive or negative outcome were similar (52% vs. 48%; *Supplementary 1*). Considering the origin of the animals, 25/39 dogs (64.10%) were shelter/stray dogs, while 10/39 (25.64%) were owned dogs.

**Figure 1:**
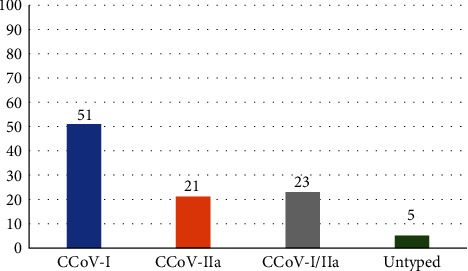
Percentages of single CCoV-I, CCoV-IIa, or mixed (CCoV-I/CCoV-IIa) coronaviral detections in the 39 positive dogs.

A total of 48 CCoV strains were detected in the 39 dogs: 29 (60.41%) of these strains were characterized as CCoV-I, 17 (35.41%) as CCoV-IIa, while two strains could not be typed (*Supplementary 1*). Single CCoV-I, CCoV-IIa, or mixed (CCoV-I and CCoV-IIa) coronaviral detections were evidenced in 20, 8, and 9 dogs, respectively (Figure 1). Overall, the prevalence of CCoV-I and CCoV-IIa in the studied population was 10.2% and 6%, respectively, while subtype CCoV-IIb was not detected.

CCoV-I strains were detected only in intestine samples or rectal swabs. CCoV-IIa strains were detected in four dogs (10.25% of the dogs that tested positive) only in intestine samples (*N* = 1) or in rectal swabs (*N* = 3), while in five dogs (12.82%), they were also found in tissue samples (lung, kidneys, liver, spleen, and mesenteric lymph nodes). Mixed (CCoV-I/CCoV-IIa) coronaviral detections were evidenced in four of these five dogs. More details are available in *Supplementary 1*.

### 3.2. CCoV Sequence and Phylogenetic Analyses

#### 3.2.1. CCoV M Gene Analysis

**Figure 2:**
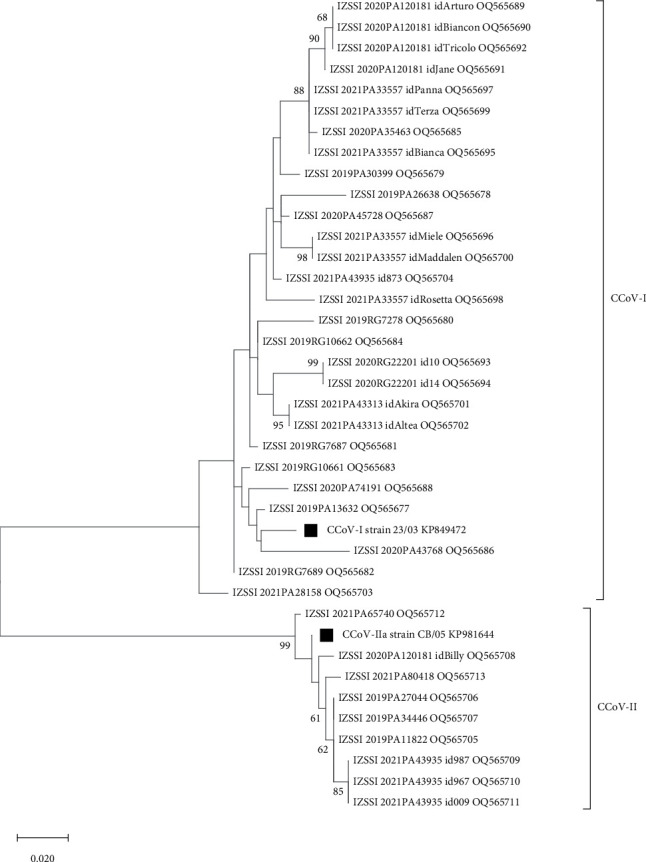
Maximum-likelihood phylogenetic tree based on 37 partial M gene sequences (370 nt) of canine coronavirus (CCoV-I and CCoV-II, as indicated) strains analysed in this study and reference CCoV strains, indicated by solid squares (▪). The phylogenetic tree was built with the maximum-likelihood method and the Tamura 3-parameter model with a discrete gamma distribution (bootstrap 1,000 replicates; bootstrap values greater than 60 are shown). Each sequence is indicated with the corresponding strain name and accession number.

All but two (dog id.s IZZSI_2019PA5124, IZSSI_2020PA120181_idNero) RT-PCR products from the screening assay were successfully sequenced. The obtained sequences (*n* = 37) were compared with CCoV-I/II reference sequences retrieved from GenBank. Twenty-eight sequences showed high nt identities (98.4%–96.2%) with CCoV-I reference strain 23/03 (accession no. KP849472), while other nine sequences had high identity (99.2%–95.7%) to CCoV-II reference strain CB/05 (KP981644). No double peaks were observed, even in coinfection cases, and only the virus with the highest load (the one with the lowest cycle threshold value; data not shown) was successfully sequenced in each of those samples. Comparison of the CCoV M gene sequences showed overall nt identities of 94.5%–100% and 97.8%–100% among CCoV-I and CCoV-II strains, respectively. Accordingly, CCoV strains were segregated into two distinct clades with reference strains CCoV-I 23/03 or CCoV-II CB/05 (Figure 2).

#### 3.2.2. CCoV-I S Gene Analysis

**Figure 3:**
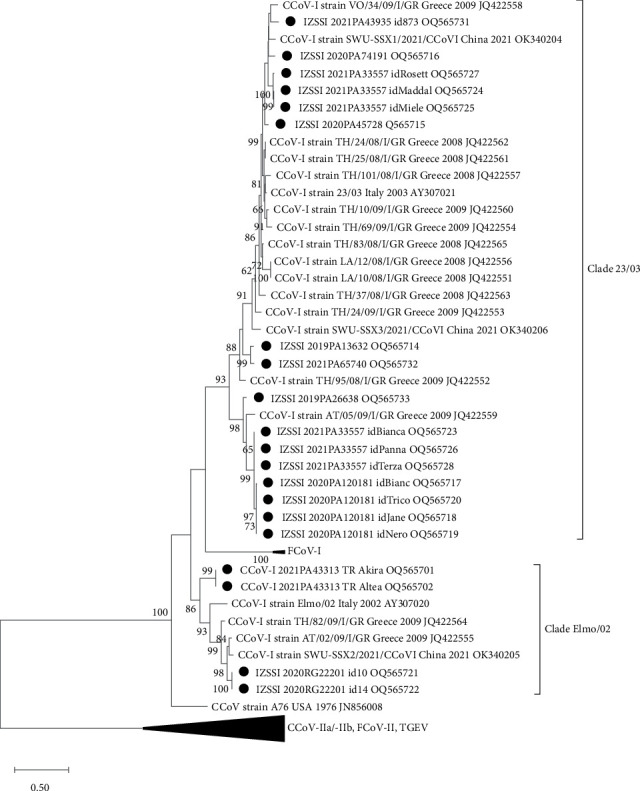
Maximum-likelihood tree based on the 5′-end of the S gene sequences (1074 nt) of CCoV-I strains analysed in this study (indicated by black dots) and reference CCoV strain. Groups including only CCoV-IIa (CCoV/NTU336/F/2008, GQ477367; 1-71, JQ404409; K378, KC175340; S378, KC175341; INSAVC, D13096; BGF10, AY342160; CB/05, DQ112226; TN-449, JQ404410), CCoV-IIb (UCD-1, AF116248; 430/07, EU924790; 174/06, EU856362; 341/05, EU856361; 119/08, EU924791), FCoV-I (Black, EU186072; UCD1, AB088222), FCoV-II (79-1146, NC007025; WSU 79-1683, JN634064), feline enteric coronavirus (79-1683, X80799), and TGEV (Purdue, DQ811789; TS, DQ201447) reference sequences are collapsed as indicated. The phylogenetic tree was built with the maximum-likelihood method and general time reversible model with a discrete gamma distribution (bootstrap 1,000 replicates; bootstrap values greater than 60 are shown). Reference CCoV-I sequences are indicated with strain name, country and year of collection, and accession number.

Good quality (based with quality score >20 for at least 95% of the length of the chromatogram) partial S sequences could be obtained for 20 out of 29 CCoV-I strains. The obtained CCoV-I sequences were 62.6%–100% nt identical to each other. A variable number (from 3 to 6) of potential glycosylation sites was predicted over 330-aa fragments, compared to the four sites observed in the 23/03 (AY307021) and Elmo/02 (AY307020) reference sequences. According to the performed phylogenetic analysis (Figure 3), five different viral clusters were observed. Specifically, viruses were grouped in two highly supported main clades (identity range between the two clades: 61.9%–67.8%) including also the two reference sequences 23/03 (within clade identities: 71.7%–100%) and Elmo/02 (75.9%–100%). Two Italian clades (total of four strains) clustered with CCoV-I strain Elmo/02, which also included strains recently identified in China and Greece, while the other three clades (16 strains) were segregated with CCoV-I strain 23/03. Interestingly, clade 23/03 comprised two divergent and highly supported subclades (identity range between the two clades: 71.7%–78.7%). The first clade (within clade identities: 80.7%–100%) also included reference strain 23/03 and several other sequences from Greece and China, while the second clade (87.4%–99.9%) was formed by eight Italian sequences and one Greek strain identified in a sample from 2009.

Strains included in both Elmo/02 and 23/03 clades were collected from outbreaks with a wide geographical distribution, which were mainly observed in 2020–2021. Interestingly, we observed distinct geographic distribution of strains. More in detail, the outbreaks involving the two Elmo/02-like strains were geographically unrelated to each other and to strains included in the 23/03 clade. Differently, strains of two different variants within the 23/03 clade (IZSSI_2021PA33557 with different id.s) originated from the same geographical area, indicating the cocirculation of multiple strains in the same place.

A similar phylogenetic tree with two main clades was obtained when we included additional shorter CCoV-I strain sequences from previous studies (*Supplementary 3*).

#### 3.2.3. CCoV-IIa S Gene Analysis

**Figure 4:**
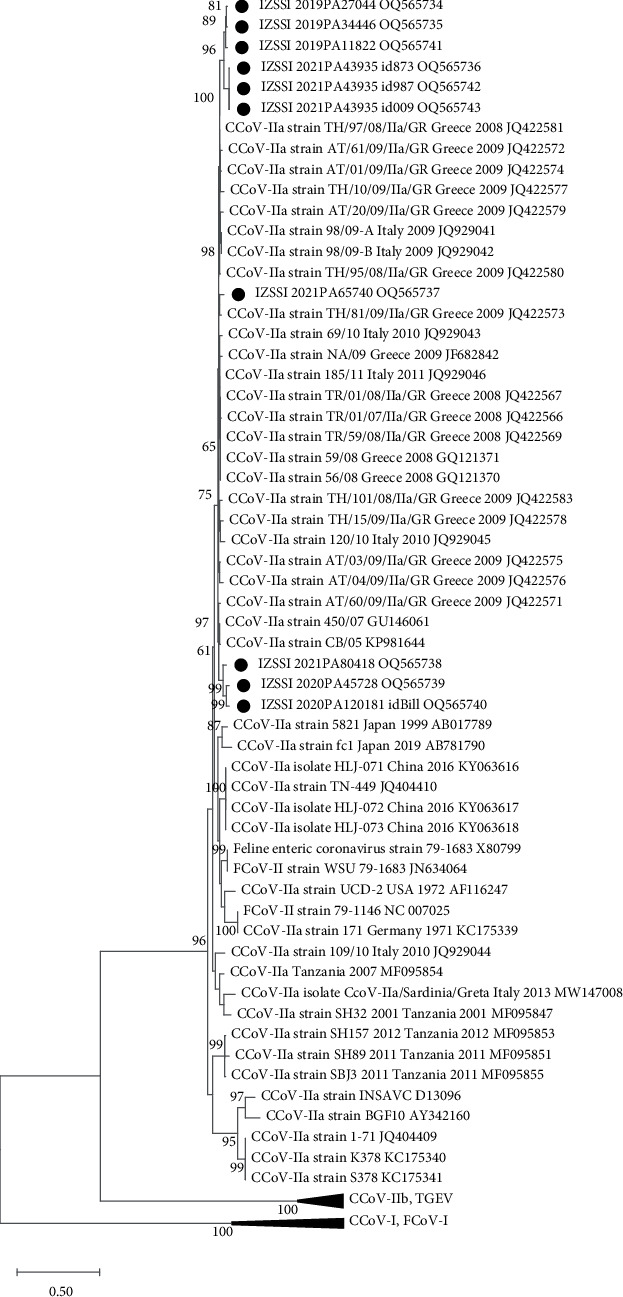
Maximum-likelihood tree based on the 5′-end of the S gene sequences (706 nt) of CCoV-IIa strains analysed in this study (indicated by black dots) and reference strains. Groups including reference CCoV-IIb (341/05, EU856361; 119/08, EU924791; CCoV/NTU336/F/2008, GQ477367; 430/07, EU924790; 174/06, EU856362; UCD-1, AF116248), TGEV (Purdue, DQ811789; TS, DQ201447), FCoV-I (Black, EU186072; UCD1, AB088222), and CCoV-I (23/03, AY307021; Elmo/02, AY307020; A76, JN856008) strains are collapsed as indicated. The phylogenetic tree was built with the maximum-likelihood method and the general time reversible model with a discrete gamma distribution (bootstrap 1,000 replicates; bootstrap values greater than 60 are shown). Reference sequences are indicated with strain name, country and year of collection, and accession number.

Partial S sequences could be successfully obtained for 10 out of 17 CCoV-IIa strains, showing an overall nt identity of 89.8%–99.1% among each other. All sequences were closely related to CCoV-IIa strains 450/07 (GU146061) and CB/05 (92.9%–95.0%) reference strains, with the highest identities being displayed with CCoV-IIa strains collected in Greece in 2008/2009 (94.3%–96.4%) and in Italy in 2009/2011 (96.4%). According to the phylogenetic analysis, CCoV-IIa strains obtained in this study belong to three different clusters, all segregating with CCoV-IIa 450/07 and CB/05 reference strains in a large clade including mainly European strains (Figure 4).

Longer S sequences (3179 nts) were also obtained for seven CCoV-IIa strains (overall nt identity of 94.6%–99.3%). A variable number (between 25 and 28) of potential glycosylation sites was predicted over 1060aa residues, compared to the 25 sites observed in the CB/05 and 450/07 reference sequences.

To include a larger number of worldwide CCoV-IIa sequences, an additional phylogenetic tree was obtained by using a dataset of shorter sequences from 132 CCoV-IIa strains detected worldwide. The strains from this study were segregated in the phylogenetic tree into a larger subclade comprising all other CCoV-II strains collected in Italy and putative pantropic CCoV-II strains, except for a few strains. Specifically, two strains (MN086812-13) collected from dogs imported to Italy from Hungary in 2017 were segregated with older European enteric CCoVs, and additionally, one strain (accession no. MF991150) collected from a wolf in 2016 and two strains from dogs imported to Italy from unknown countries in 2016 (MN086812-13, MN086803, and MN086805) were segregated in a close subclade along with CCoV-II strains from Asian countries (*Supplementary 4*).

## 4. Discussion

CCoVs are widespread in domestic dogs, being the causative agents of mild gastroenteritis, characterized by high morbidity and low mortality, while some variants are associated with systemic and often fatal disease [1]. CCoVs cause disease alone or, more often, in coinfection with other viral pathogens, impacting the health of canines worldwide [35]. CCoV has also been reported as the causative agent of disease in different wild species of the order *Carnivora* [17, 31, 36], but host specificity among wild animals has not been fully determined yet.

The recent emergence of SARS-CoV-2, along with the detection of clinical disease in humans associated with distinct CCoV variants [37, 38], further highlights the key role of animals, including companion animals, as reservoirs or intermediate hosts of coronaviruses transmissible to humans [4, 37, 39]. Additionally, recombination events have been documented between CCoV types as well as among closely related viruses, such as FCoV or TGEV, resulting in new virulent coronavirus strains [35]. This indicates the need for constant animal coronavirus surveillance, especially in those animals that have frequent contacts with humans.

In the past 15 years, enteric CCoVs have been occasionally reported in Italy, in both domestic dogs [6, 18, 19] and wolves [17], as well as in domestic dogs in other European countries [15, 16, 27, 29]. Despite the recently renewed interest in enteric CCoVs, available sequences and information on the molecular epidemiology and genomic features of strains circulating in Europe, particularly with regard to CCoV-I, are still limited. The aim of this study was to provide an in-depth update on the epidemiological and molecular features of CCoVs identified in owned, shelter/kennel, and stray dogs in Italy.

CCoV RNA was detected in 13.7% of sampled dogs, and most of the positive dogs were 2 months old or younger. The positivity rate was lower compared to those reported in other regions of southern Italy, such as Sardinia (22.2%) [19] and Campania (31.1%) [40]. CCoV was detected in older dogs in only eight cases, mostly (*n* = 6) in coinfection with other viruses. Overall, most of the CCoV-positive dogs presented coinfections with other enteric viruses, with CPV-2a/-2b/-2c being the most frequently detected agent (84.6%). Coinfections with neglected (CAdV-1) or potentially zoonotic (NoV) viruses were also observed, although at low rates. These data confirm that CCoV mostly affects puppies or young dogs [41] and that it is often found in coinfections [19], particularly with CPV-2. While similar proportions of positive or negative outcome (52% vs. 48%) were observed for dogs that survived, who almost always tested positive for at least two different viruses, a higher CCoV positivity rate was observed among shelter/stray dogs (64.10%) compared to owned dogs (25.64%). This suggests the potential role of other factors, such as vaccinations or environmental stressors, in favouring CCoV diffusion or persistence, as observed in other studies [40, 42, 43].

CCoV-I was detected with a somewhat higher prevalence (10%) compared to CCoV-II (6.0%) and as the only genotype in most single CCoV infections (20/28 dogs). Among CCoV-II-positive dead dogs (*n* = 9), this viral genotype was detected only in the intestinal tract of five dogs and in the intestine and other tissues from the remaining four dogs. The positivity rate for pantropic CCoV was lower (2.99%) than in a previous study (9.94%) [18]. Due to the type of sample available (rectal swab) for other eight dogs, it was not possible to determine whether systemic spread occurred. Nonetheless, CCoV-I remains the main detected type in this study, in agreement with other currently available epidemiological studies [29, 35]. Further data are necessary to better evaluate the spread of this viral type, compared to CCoV-II, also including the putative pantropic mutants. Since CCoV-I cannot be cultured *in vitro* and, more generally, CCoVs are difficult to isolate in cell cultures [16, 26, 44], limiting the possibility of carrying out studies on CCoV-I pathogenicity, particularly in CCoV-I/II mixed infections [29], sequence analysis of currently circulating CCoV strains, coupled with epidemiological and pathological investigations, could help to shed some light on pathological aspects of CCoVs.

Among the targets analysed in this study, the M gene was suitable for CCoV detection even at low RNA concentrations, as also demonstrated by the results obtained with qPCR (data not shown). However, while it allowed us to distinguish, after sequencing the amplified product, between CCoV types in dogs with single CCoV infection, the screening PCR we used allowed the characterization of the prevailing viral type in coinfections, preventing to identify mixed CCoV infections. The latter were detected by using specific typing molecular assays, confirming the need for multiple tests specific for the screening, detection, and/or sequencing of CCoV strains.

Even if a low number of CCoV-I sequences are currently available in GenBank, limiting our conclusions, the results we obtained allowed us some useful considerations. Based on a large and informative S sequence of both CCoV types, including the NTD, a higher variability among CCoV-I strains was observed in comparison to CCoV-II (62.6%–100% vs. 89.8%–99.1% and 5 vs. 3 clusters). This was also true when comparing CCoV-I to reference (European or Chinese) strains. The higher divergence among CCoV-I strains, especially with regard to older reference strains, suggests that viral diversity could be higher than previously thought and further highlights the need to obtain additional sequence information and to collect more epidemiological data to properly assess the current distribution and diversity of CCoV-I.

Conversely, higher nt identities among sequenced CCoV-IIa strains and between these and reference strains collected in Europe, as well as the lack of detection of CCoV-IIb strains, suggest a lower CCoV-II diversity in Italy, at least for what concerns strains sequenced in the past fifteen years. Most of the CCoV-IIa strains included in this large group were considered as pantropic, being detected in one or more extraintestinal tissues [16, 18, 30]. Among the dead dogs evaluated in the present study, the proportion of animals that presented CCoV-IIa in the intestinal tract alone was similar to that of animals whose extra-intestinal tissues were also infected. However, none of the previously described genomic markers characterizing pantropic strains, including the S-125 aa residue [30], was observed among our sequences. This may indicate the potential presence of additional yet undetected markers which could be responsible for the change in tissue tropism.

Analysed CCoV-II strains clustered in a subclade separate from other Italian sequences obtained from dogs illegally imported from unknown countries [18], which were included in a large phylogenetic subclade including mostly Asian strains. Intriguingly, this clade did not include any other Italian sequence except for the only CCoV-IIa strain from an autochthonous wild carnivoran [17]. This raises questions about the potential origin and distribution of Asian CCoV strains. However, the local viral diversity may be currently underestimated given the low number of studies that molecularly characterized CCoV in Italy and elsewhere. For CPV-2c, the long-distance spread of Asian strains among European and North American domestic dogs and wild carnivorans was observed [45–48]. As it is possible that CCoV spread in a similar way, further studies are crucial to elucidate where these strains originated and whether they emerged in domestic dogs and then spread to wild animals or vice versa. Even if longer sequences could better define currently undetected genomic divergence between European and Asian strains, the genome analysis of the NTD within the S1 subunit performed in this study still allowed to assess the genetic relationship among CCoV strains and, thus, preliminarily study viral movements.

The role of enteric coronaviruses as significant canine pathogens and the value of vaccination against CCoVs still remain controversial [49, 50], so much so that international guidelines do not recommend the use of CCoV vaccines [50] or their use is not considered [51]. However, experimental infections demonstrated the ability of hypervirulent CCoV types to induce systemic disease with acute lymphopenia [52] or subsequent prolonged depletion of peripheral CD4+ T cells, which theoretically may lead to a potential exposure to opportunistic infections [53]. Considering the high rates of CCoV/CPV-2 mixed infections, which were also observed in this study, and the higher significance of CPV-2 variants as canine enteropathogen, efforts should be better directed towards the control of CPV-2a/-2b/-2c infections [51] in order to contribute to the general canine welfare, reduce the overall incidence of viral diseases in domestic dogs, and prevent spillover to wildlife. However, given the variable pathogenic potential of the different strains, we underline the need to further investigate the epidemiology and evolution of CCoVs and be better prepared to face the emergence of novel genetic lineages, which could also impact human health.

Due to the retrospective nature of the study and the unavailability of some qualitative data or specific tests (e.g., histological/immunohistochemical evaluation), some clinical and epidemiological aspects could not be evaluated in detail. Another limitation of this study was that the full viral genomes were not obtained as sequencing was limited to a large, although not complete, portion of the spike gene. This prevented the identification of recombinant viruses and the evaluation of other genomic regions that might be important for virulence determination.

## 5. Conclusions

In conclusion, this study highlights the importance of epidemiologic surveys for coronaviruses in domestic dogs, here spanning a temporal period overlapping the COVID-19 pandemic. Based on these results, CCoV is widespread in Italy, with the cocirculation (also in the same area) of both genotypes and several different clades. Young dogs had the highest rates of CCoV infection, and mixed infection with other enteric viruses was frequently observed, with CCoV-I type having a higher prevalence and genetic variability than CCoV-II. Our data contributed to expanding the knowledge on CCoV epidemiology, suggesting that additional studies are required to further determine the genomic heterogeneity of CCoV and its pathogenic potential in domestic dogs.

## Data Availability

Sequences included in this study have been submitted to the DDBJ/EMBL/GenBank databases under accession numbers OQ565677–OQ565713 (gene M) and OQ565714–OQ565743 (gene S).

## Supplementary Materials

Supplementary 1 Details on age, origin, health status and clinical outcome, viral genotype, and viral coinfections for each CCoV-positive dog.

Supplementary 2 Sequences and positions of the primers used in gel-based RT-PCR assays of the present study.

Supplementary 3 Maximum-likelihood tree based on the 5′-end of the S gene sequences (364 nt) of CCoV-I strains analysed in this study and reference CCoV strains.

Supplementary 4 Maximum-likelihood tree based on the 5′-end of the S gene sequences (369 nt) of CCoV-II strains analysed in this study and reference CCoV strains.
