# Supplementary material for: Molecular Screening and Characterization of Canine Coronavirus Types I and II Strains from Domestic Dogs in Southern Italy, 2019–2021
Source: Transbound Emerg Dis. 2024 Apr 18;2024:7272785. doi: 10.1155/2024/7272785 (PMC12016997; doi:10.1155/2024/7272785)
Supplement: Supplementary 1 — Details on age, origin, health status and clinical outcome, viral genotype, and viral coinfections for each CCoV-positive dog. [file 7272785.f1.docx]

**Supplementary Table S1.** Details on age, origin, health status, clinical outcome (in brackets), viral genotype, and viral co-infections for each CCoV-positive dog.

| **Identifier** | **Age** | **Origin** | **Status**  **(outcome)** | **Positive samples** | **Genotype** | **Co-infections^1^** |
| --- | --- | --- | --- | --- | --- | --- |
|  |  | | | | | |
|  | **2019** | | | | | |
| **IZSSI_2019RG7278** | 2 months | shelter | sick  (positive) | Rectal swab | CCoV-I | CPV-2c, Norovirus GIV.2 |
| **IZSSI_2019RG7687** | 2 months | shelter | sick  (positive) | Rectal swab | CCoV-I | CPV-2c |
| **IZSSI_2019RG7689** | 2 months | shelter | sick  (positive) | Rectal swab | CCoV-I | CPV-2c |
| **IZSSI_2019RG10661** | 2 months | owned | sick  (positive) | Rectal swab | CCoV-I | CPV-2c |
| **IZSSI_2019RG10662** | 2 months | shelter | sick  (negative) | Rectal swab | CCoV-I | CPV-2c |
| **IZSSI_2019PA30399** | 2 months | owned | sick  (positive) | Rectal swab | CCoV-I | CPV-2c |
| **IZSSI_2019PA5124** | 2 months | owned | dead | Intestine, spleen, mesenteric  lymph nodes | Undet.^2^ | CPV-2a |
| **IZSSI_2019PA11822** | 2 years | stray | dead | Intestine, lungs, kidneys, liver | CCoV-I, - IIa | CPV-2c, CAdV-1 |
| **IZSSI_2019PA13632** | 6 months | stray | dead | Intestine | CCoV-I | CPV-2a |
| **IZSSI_2019PA26638** | 9 months | imported | dead | Intestine | CCoV-I | CPV-2a |
| **IZSSI_2019PA27044** | 5 months | stray | dead | Intestine | CCoV- IIa | CPV-2a |
| **IZSSI_2019PA34446** | 11 months | stray | dead | Intestine, lungs | CCoV- IIa | CAdV-1 |
|  |  | | | | | |
|  | **2020** | | | | | |
| **IZSSI_2020PA43768** | 2 months | shelter | sick  (positive) | Rectal swab | CCoV-I | CPV (undet.^1^) |
| **IZSSI_2020PA74191** | 2 months | owned | sick  (negative) | Rectal swab | CCoV-I | -^3^ |
| **IZSSI_2020PA120181_idArturo** | 2 months | owned | sick  (positive) | Rectal swab | CCoV- IIa | CPV-2b |
| **IZSSI_2020PA120181_idBill** | 2 months | owned | sick  (positive) | Rectal swab | CCoV- IIa | CPV-2c |
| **IZSSI_2020PA120181_idJane** | 2 months | shelter | sick  (positive) | Rectal swab | CCoV-I, - IIa | CPV-2b |
| **IZSSI_2020PA120181_idNero** | 2 months | shelter | sick  (negative) | Rectal swab | CCoV-I | CPV-2b |
| **IZSSI_2020PA120181_idBianc** | 2 months | shelter | sick  (negative) | Rectal swab | CCoV-I, - IIa | CPV-2b |
|  |  |  |  |  |  |  |
| **IZSSI_2020PA120181_idTrico** | 2 months | shelter | sick  (positive) | Rectal swab | CCoV-I, - IIa | CPV-2b |
| **IZSSI_2020RG22201_id10** | 2 months | kennel | sick  (negative) | Rectal swab | CCoV-I, - IIa | CPV-2 |
| **IZSSI_2020RG22201_id14** | 2 months | kennel | sick  (negative) | Rectal swab | CCoV-I, - IIa | -^3^ |
| **IZSSI_2020PA35463** | 40 days | owned | dead | Intestine | CCoV-I | -^3^ |
| **IZSSI_2020PA45728** | 5 years | owned | dead | Intestine, kidneys, lungs | CCoV-I, - IIa | -^3^ |
|  |  | | | | | |
|  | **2021** | | | | | |
| **IZSSI_2021PA33557_idBianca** | 2 months | shelter | sick  (negative) | Rectal swab | CCoV-I | CPV-2c |
| **IZSSI_2021PA33557_idMaddalen** | 2 months | shelter | sick  (negative) | Rectal swab | CCoV-I | CPV-2c, Norovirus GIV.2 |
| **IZSSI_2021PA33557_idMiele** | 2 months | shelter | sick  (positive) | Rectal swab | CCoV-I | CPV-2c, Norovirus GIV.2 |
| **IZSSI_2021PA33557_idPanna** | 2 months | shelter | sick  (negative) | Rectal swab | CCoV-I | CPV-2c |
| **IZSSI_2021PA33557_idRosetta** | 2 months | owned | sick  (negative) | Rectal swab | CCoV-I | CPV-2c |
| **IZSSI_2021PA33557_idTerza** | 2 months | shelter | sick  (positive) | Rectal swab | CCoV-I | CPV-2c |
| **IZSSI_2021PA43313_idAkira** | 2 months | owned | sick  (negative) | Rectal swab | CCoV-I | CPV-2c |
| **IZSSI_2021PA43313_idAltea** | 2 months | owned | sick  (negative) | Rectal swab | CCoV-I | CPV-2c |
| **IZSSI_2021PA80418** | 2 months | shelter | sick  (positive) | Feces/Rectal swab | CcoV- IIa | CPV-2c |
| **IZSSI_2021PA28158** | 12 months | shelter | dead | Mesenteric lymph nodes | Undet.^2^ | CPV-2c |
| **IZSSI_2021PA43935_id873** | 2 months | shelter | dead | Intestine | CCoV-I, - IIa | CPV-2b |
| **IZSSI_2021PA43935_id967** | 2 months | shelter | dead | Intestine | CCoV-II | CPV-2b |
| **IZSSI_2021PA43935_id987** | 2 months | shelter | dead | Intestine, lungs | CCoV-II | CPV-2b |
| **IZSSI_2021PA43935_id009** | 2 months | shelter | dead | Intestine | CCoV-II | CPV-2b |
| **IZSSI_2021PA65740** | 11 years | shelter | dead | Intestine, mesenteric  lymph nodes | CCoV-I, - IIa | -^3^ |

^1^CPV-2 original type or variants (CPV-2a, CPV-2b, CPV-2c); ^2^genotype undetermined; ^3^none.
