# Supplementary material for: Molecular Screening and Characterization of Canine Coronavirus Types I and II Strains from Domestic Dogs in Southern Italy, 2019–2021
Source: Transbound Emerg Dis. 2024 Apr 18;2024:7272785. doi: 10.1155/2024/7272785 (PMC12016997; doi:10.1155/2024/7272785)
Supplement: Supplementary 2 — Sequences and positions of the primers used in gel-based RT-PCR assays of the present study. [file 7272785.f2.docx]

**Supplementary Table S2.** Sequences and positions of the primers used in gel-based RT-PCR assays of the present study.

| **Primer** | **Gene** | **CCoV type** | **Sequence (5’-3’)** | **Strand** | **Annealing**  **Temp (°C)** | **Amplicon**  **size (bp)** | **Reference** |
| --- | --- | --- | --- | --- | --- | --- | --- |
| CCV1^a^ | M | I/II | TCCAGATATGTAATGTTCGG | + | 55°C | 409 | [14] |
| CCV2^a^ |  |  | TCTGTTGAGTAATCACCAGCT | - |  |  |  |
| CCoV1a^b^ | M | I | GTGCTTCCTCTTGAAGGTACA | + | 55°C | 239 | [26] |
| CCoV2^b^ |  |  | TCTGTTGAGTAATCACCAGCT | - |  |  |  |
| Can1F^b^ | M | II | TAACATTGCTCTCAGGGAATTTG | + | 55°C | 202 |  |
| CCoV2^b^ |  |  | TCTGTTGAGTAATCACCAGCT | - |  |  |  |
| EL1F^b^ | S | I | CAAGTTGACCGTCTTATTACTGGTAG | + | 55°C | 346 |  |
| EL1R^b^ |  |  | TCATATACGTACCATTATAGCTGAAGA | - |  |  |  |
| S5^b,c^ | S | IIa | TGCATTTGTGTCTCAGACTT | + | 55°C | 694 |  |
| S6^b,c^ |  |  | CCAAGGCCATTTTACATAAG | - |  |  |  |
| CEPol-1^b,c^ | ORF1b | IIb | TCTACAATTATGGCTCTATCAC | + | 50°C | 370 | [27] |
| TGSP-2^b,c^ | S |  | TAATCACCTAAMACCACATCTG | - |  |  |  |
| 20179^c,e^ | ORF1b  S | IIa/IIb | GGCTCTATCACATAACTCAGTCCTAG | + | 55°C | 760 (CCoV-IIa)  498 (CCoV-IIb) | [15] |
| INS-R-dg^c,e^ |  | IIa | GCTGTAACATAKTCRTCATTCCAC | - |  |  |  |
| 174-268^c,e^ |  | IIb | CAACATGTAACCTTTGTCTGTGATCTGC | - |  |  |  |
| PolEnd^d^ | ORF1b | I | CTAAGGAAGGGTAAGTTGCTCA | + | 52°C | 1000 | [29] |
| 1060dR^d^ | S |  | ATCAGCAGATGCTTGRGGACA | - |  |  |  |
| El-Ins1^e^ | S | IIa | GGATTACTAARGADKGGTAAGTTGC | + | 50°C | 1631 | [30] |
| S2^e^ |  |  | CAACTTCACTTGAAGCAACA | - |  |  |  |
| SIIF^e^ |  |  | GCATTAGTAGTTGAAAACACAGC | + | 50°C | 1285 |  |
| SIIR^e^ |  |  | TCAAGTCTGGCACCCATTG | - |  |  |  |
| S3^e^ |  |  | GAGACTTTCAACCAATTAGC | + | 50°C | 1876 |  |
| UCD6R^e^ |  |  | TTAATGAATGTGAACTTTTTCAATAGG | - |  |  |  |
| SF1^e^ | S | II | ATGATTGTGCTTACATTGTGCC | + | 50°C | 1640 | [31] |
| SR1^e^ |  |  | CCACTACGCTTCATACCAAGAT | - |  |  |  |
| SF2^e^ |  |  | TCTTGGTATGAAGCGTAGTGG | + | 50°C | 1613 |  |
| SR2^e^ |  |  | TACCAATAGCTTGATTGAAAGC | - |  |  |  |

Primers used for ^a^CCoV screening, ^b^CCoV I/II typing, ^c^CCoV-IIa and CCoV-IIb subtyping, and ^d^CCoV-I or ^e^CCoV-II 5’-S gene sequencing.
