# Supplementary figures and images for: Molecular Screening and Characterization of Canine Coronavirus Types I and II Strains from Domestic Dogs in Southern Italy, 2019–2021
Source: Transbound Emerg Dis. 2024 Apr 18;2024:7272785. doi: 10.1155/2024/7272785 (PMC12016997; doi:10.1155/2024/7272785)

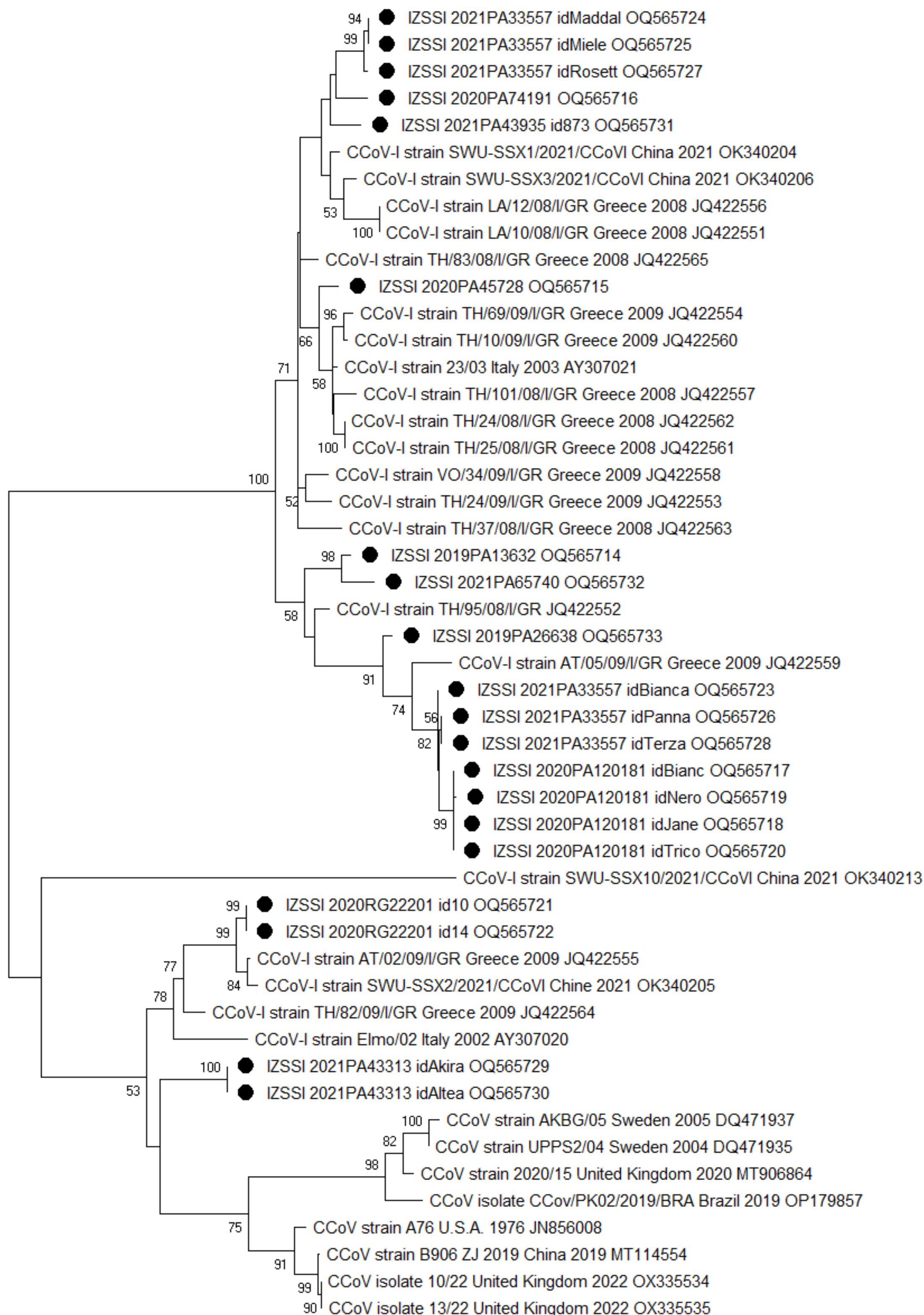

0.20

Supplement: Supplementary 3 — Maximum-likelihood tree based on the 5′-end of the S gene sequences (364 nt) of CCoV-I strains analysed in this study and reference CCoV strains. [file 7272785.f3.pdf]

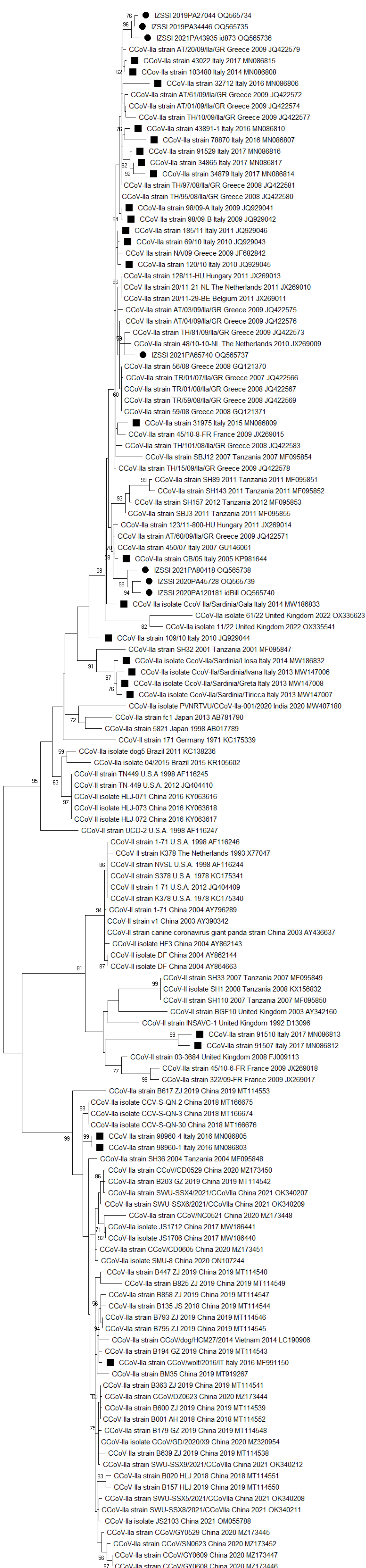

0.050

Supplement: Supplementary 4 — Maximum-likelihood tree based on the 5′-end of the S gene sequences (369 nt) of CCoV-II strains analysed in this study and reference CCoV strains. [file 7272785.f4.pdf]
